# A Time to Rest, a Time to Dine: Sleep, Time-Restricted Eating, and Cardiometabolic Health

**DOI:** 10.3390/nu14030420

**Published:** 2022-01-18

**Authors:** Charlotte C. Gupta, Grace E. Vincent, Alison M. Coates, Saman Khalesi, Christopher Irwin, Jillian Dorrian, Sally A. Ferguson

**Affiliations:** 1 Appleton Institute, Central Queensland University, Adelaide 5034, Australia; g.vincent@cqu.edu.au (G.E.V.); sally.ferguson@cqu.edu.au (S.A.F.); 2 Alliance for Research in Exercise, Nutrition and Activity (ARENA) Research Centre, University of South Australia, Adelaide 5001, Australia; alison.coates@unisa.edu.au; 3 Behaviour-Brain-Body Research Centre, UniSA Justice and Society, University of South Australia, Adelaide 5072, Australia; jill.dorrian@unisa.edu.au; 4 Appleton Institute, Central Queensland University, Brisbane 4000, Australia; s.khalesi@cqu.edu.au; 5 School of Health Sciences and Social Work, Griffith University, Gold Coast 4222, Australia; c.irwin@griffith.edu.au

**Keywords:** chrono-nutrition, meal timing, eating habits, metabolic health, cardiovascular, sleep timing, circadian disruption, night shift

## Abstract

Cardiovascular disease (CVD) poses a serious health and economic burden worldwide. Modifiable lifestyle factors are a focus of research into reducing the burden of CVD, with diet as one of the most investigated factors. Specifically, the timing and regularity of food intake is an emerging research area, with approaches such as time-restricted eating (TRE) receiving much attention. TRE involves shortening the time available to eat across the day and is associated with improved CVD outcomes compared with longer eating windows. However, studies that have examined TRE have not considered the impact of sleep on CVD outcomes despite recent evidence showing that sleep duration can influence the timing and amount of food eaten. In this article, we argue that as TRE and sleep influence each other, and influence the same cardiometabolic parameters, experiencing inadequate sleep may attenuate any positive impact TRE has on CVD. We examine the relationship between TRE and CVD, with sleep as a potential mediator in this relationship, and propose a research agenda to investigate this relationship. This will provide necessary evidence to inform future interventions aimed at reducing the burden of CVD.

## 1. Introduction: Cardiometabolic Risk and Chronobiology

Cardiovascular disease (CVD) is an umbrella term used to describe medical conditions that affect the heart, blood vessels, and cardiometabolic health [1,2]. CVD is the leading cause of death globally [3,4] and, importantly, is largely preventable [5]. Modifiable lifestyle factors, including sub-optimal diet, physical inactivity, excessive alcohol consumption, and smoking, account for up to 90% of the risk factors associated with CVD [2].

The Heart Foundation Australia [2] emphasises a need to focus on modifiable lifestyle factors as part of future research into reducing the burden of CVD. When investigating how modifiable lifestyle factors influence CVD, research to date has focused on factors such as diet, physical activity, smoking, and alcohol consumption [1,5,6,7]. However, as the rates of CVD continue to grow [2,4], the focus of research has widened to include other lifestyle factors that could also be important contributors to CVD risk, such as sleep [8,9,10]. Attention has also turned to exploring possible interactions between lifestyle factors to identify novel approaches to reducing CVD risk. While sub-optimal diet is a well-established risk factor for CVD [11,12,13], a new focus is emerging on the timing and regularity of food intake [14,15,16]. Given that the timing and regularity of sleep is also a consideration in relation to CVD risk factors [8,17], the next step is to explore the timing of both sleep and food intake, alongside our expanding understanding of biological timing systems.

Identifying the mechanisms underlying CVD is integral to reducing the burden of disease [18,19]. Disruption to our circadian rhythms is one such mechanism that has received scientific attention, with several key reviews demonstrating the relationship between circadian disruption and cardiometabolic health [9,18,20,21]. Circadian rhythms are biological and behavioural rhythms with a period of approximately 24 h [22,23]. While it is well-established that the circadian system, controlled by a central clock located in the suprachiasmatic nucleus in the brain, is influenced by light [24], more recent evidence demonstrates that peripheral clocks throughout our body are influenced by other external behaviours, such as meal timing [25,26]. Furthermore, while sleep timing affects light exposure and therefore the central clock, the timing of sleep has also been linked to the timing of peripheral clocks [18].

Optimal functioning of the circadian system is essential for good health [27]. Circadian disruption occurs when our sleep–wake and eating–fasting rhythms are not appropriately timed; that is, they do not align with our light–dark cycle [18,28]. One of the leading causes of circadian disruption is working non-standard hours including shift work [9]. Shift work disrupts the usual sleep–wake cycle as workers may be on shift during times when the body is primed to be sleeping (i.e., at night), attempting sleep when the body is primed to be awake (i.e., during the day), or woken during a sleep period when working an on-call schedule [29,30]. This leads to inadequate sleep in shift work populations [31,32], contributing to circadian disruption, which is recognised as a major contributor to CVD risk in shift workers [9]. As a result of circadian disruption, natural physiological processes, such as metabolism, digestion, energy expenditure, and blood pressure, are misaligned [9,20,23,24,26,33,34,35,36,37,38,39]. This misalignment is proposed to play a critical role in the development of long-term health problems, such as CVD [1,9].

Eating at a time when our body is not primed to digest food is a challenge to multiple physiological systems, including the circadian system [14]. The typical eating window (the time from first to last time of energy consumption across the day) spans 14 h of the day in healthy, synchronised individuals [40,41]. Spreading eating events across the day (i.e., beyond the 14 h eating window) to include eating late at night is associated with weight gain and increased insulin resistance [42], which are two markers of CVD. Importantly, the typical eating window may be longer in obese populations [43]. In response to these novel findings, time-restricted eating (TRE), whereby the eating window is shortened [44], has been proposed to manage weight and cardiometabolic health. Indeed, several recent studies investigating TRE and cardiometabolic health [14,45,46,47,48,49,50,51] have demonstrated improved cardiometabolic outcomes with TRE, including reductions in systolic and diastolic blood pressure, reductions in fat mass, and improved insulin sensitivity [50,51,52]. While some of these studies measured sleep as an outcome after a TRE intervention [53], they did not consider sleep as a predictor of cardiometabolic health outcomes and therefore missed the opportunity to control for or to consider the impact of chronic inadequate sleep. This is potentially problematic as circadian disruption resulting from inadequate sleep may independently influence the same cardiovascular outcomes influenced by TRE [8,54]. Furthermore, there is a known relationship between food intake and sleep [55,56,57] such that inadequate sleep can lead to altered meal timing [55,58] and increased cravings for certain foods [59,60], and different foods and nutrients can impact sleep quality [61,62]. These interactions highlight the need to better understand the relationships between eating patterns, such as TRE, sleep, circadian misalignment, and cardiometabolic health.

In this review, these relationships are proposed to follow a mediator model, recognising that inadequate sleep may partially mediate the relationship between TRE and cardiometabolic outcomes. The relationship between cardiometabolic health and TRE will be discussed, followed by the relationship between cardiometabolic health and sleep in the context of the timing of eating. It will be argued that sleep mediates the relationship between TRE and improved cardiometabolic outcomes, and an agenda for a proposed program of research will be outlined.

## 2. Time-Restricted Eating, Circadian Disruption, and Cardiometabolic Health

Eating habits are a well-researched lifestyle factor contributing to CVD [63]. Poor eating habits, such as poor diet quality, have been linked to key cardiometabolic risk factors for CVD [11,12,64,65], including increased blood pressure, body mass index, serum lipids, total cholesterol, and low-density lipoprotein cholesterol [12,66,67]. As such, improving eating habits is a critical part of reducing CVD risk [13,68]. However, it has become apparent that the timing of food intake is also an important consideration [38], primarily due to the burden of altered eating patterns on the circadian system [14].

Eating at times when our body is not primed to digest food (e.g., at night [16]) can compromise metabolism and thus lead to an increase in the likelihood of developing CVD [38,44]. Indeed, observational studies have reported a relationship between irregular eating patterns and increased risk for metabolic syndrome [69,70,71], in addition to a relationship between habitual night eating and arterial stiffness, a preclinical sign of CVD [72]. Altered eating patterns are characteristic of shift work [55,73,74,75], with shift workers reporting that they change the timing of eating to accommodate their shift schedule; for example, eating during the night when working night shifts [55]. Misalignment of eating rhythms with the internal circadian system is thought to contribute to the relationship between shift work and CVD [73]. Research in non-shift workers has shown that erratic eating patterns are associated with increased CVD risk after controlling for dietary composition [76]; thus, highlighting that in addition to ‘what is eaten’, ‘when eating occurs’ also influences CVD risk. Collectively, recent research highlights the need to optimise eating time to reduce circadian disruption and consequently reduce the prevalence of CVD.

**Figure 1:**
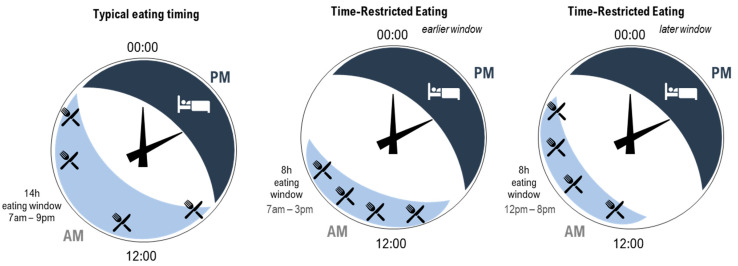
Illustration of three patterns of eating (light blue shading with knife and fork) and sleeping (dark blue shading with bed) across hours on 24 h clocks. **Left**—typical eating arrangement within a 14 h eating window starting at 7 am. **Middle**—time-restricted eating within an 8 h eating window starting at 7 am. **Right**—time-restricted eating within an 8 h eating window starting at 12 pm. All arrangements include the same number of eating occasions (indicated by knife and fork). For the time-restricted eating patterns, the time between the first and last eating occasion is shortened, increasing the length of the overnight fast.

As previously discussed, TRE is a unique strategy that has gained popularity as a way to optimise the timing of food intake [14,16,38]. While typical eating windows for most individuals span 14 h of the day, a TRE approach shortens the eating window to between 4 and 10 h [14,77] (Figure 1, example 8 h eating window). It is important to note that the optimal timing of a shortened eating window (i.e., starting the eating window early in the day or later in the day) requires systematic study [44]. In a recent review by Regmi and Heilbronn [78], the authors argued that while the early morning (e.g., Figure 1, middle panel) may be considered an optimal time to start the eating window for maximal metabolic benefits (e.g., improving insulin sensitivity and lipid absorption), this would mean that people would miss eating dinner at a traditional time (6–8 pm), which is a typical family and group eating time [79]. This may therefore present social challenges. In contrast, the same 8 h eating window could start at 12 pm and include the typical dinner time (e.g., Figure 1, right panel). While this arrangement extends the overnight fast to the same degree and may be perceived to be less socially challenging, metabolic benefits may be reduced due to circadian considerations [78].

TRE may lead to improved cardiovascular outcomes [16]. For example, a recent systematic review and meta-analysis of eleven studies found significantly lower fasting glucose values for participants on a TRE pattern (with eating windows ranging from 6 h to 12 h) compared to those eating ad libitum [50]. Other studies have similarly demonstrated improvements following TRE in blood pressure, body weight, cholesterol, glucose metabolism, and the gut microbiome [22,50,51,78,80,81]. Furthermore, TRE circumvents some of the challenges of typical dieting approaches, which often require individuals to employ restrictive behaviours, because the quality and quantity of the food eaten in TRE regimes does not change [77,82], as only the window of eating is altered.

**Figure 2:**
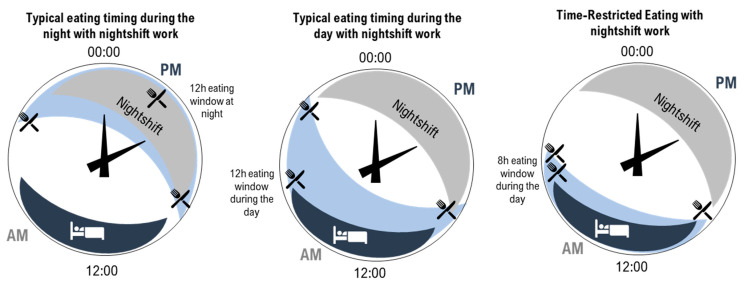
Illustration of three patterns of eating (light blue shading with knife and fork) and sleeping (dark blue shading with bed) on 24 h clocks. **Left**—typical eating arrangement for a night-shift worker with a 12 h eating window during the night. **Middle**—example of an eating arrangement for a night-shift worker with a 12 h eating window during the day. **Right**—time-restricted eating for a night-shift worker with an 8 h eating window during the day (right).

In shortening the eating window, TRE reduces the amount of time the body is required to metabolise food and lengthens the daily fast period, arguably allowing for greater metabolic recovery [14,16,22,41,50]. As introduced in Figure 1, a further opportunity for improving cardiometabolic outcomes arises from the ability to consider the timing of the eating window to reduce circadian disruption. In people with typical diurnal rhythm, starting a shortened eating window early in the day avoids circadian misalignment of eating behaviours and related impairment in the way in which food is processed by the body [15,38]. Indeed, evidence suggests that an earlier eating window is associated with more effective cardiometabolic outcomes compared to a later eating window [14,16,41,77]. As previously discussed, circadian disruption plays a significant role in the development of CVD due to misaligned daily rhythms, such as metabolism, digestion, and blood pressure [1]. Since food acts as a signal for peripheral circadian clocks [16,18], eating food at biologically inappropriate times can lead to misalignment between central and peripheral clocks [83]. Therefore, carefully timed TRE may be an effective and relatively straightforward strategy to minimise circadian disruption [48] and ultimately contribute to a reduced burden of CVD [38,77]. However, while day interventions are the focus of much of the existing TRE literature [14,38,45,50,84], consideration of the impacts of practicing TRE at other times, such as during the night, has been less well-documented. Research in this area is of particular interest for night workers who typically eat during the night [55]. Recent laboratory research has demonstrated the beneficial effect for glucose metabolism of maintaining a daytime eating window even while working (simulated) night shifts [85,86,87,88]. To extend this research, additional consideration of TRE for those working night shifts is needed; in particular, this would determine the impact and feasibility of shortening the daytime eating window while supporting the need to sleep during the day (Figure 2).

Consideration of sleep in the context of night work is critical. While TRE can be designed to reduce circadian disruption, one of the biggest contributors to circadian disruption is an altered sleep–wake cycle [23,26]. We hypothesise that TRE may not have the same benefits for cardiometabolic health if individuals are experiencing circadian disruption due to inadequate sleep.

## 3. Inadequate Sleep, Circadian Disruption, and Cardiometabolic Health

Inadequate sleep is highly prevalent globally, with adults commonly obtaining less than the optimal 7–9 h of sleep per night [89,90,91,92,93]. For example, up to one-third of Australians do not achieve 7 h of sleep per night [89,90]. This is problematic, as chronic inadequate sleep challenges the circadian system [19] and consequently impacts cardiometabolic health [1,10,19,94]. Several studies have demonstrated the link between short sleep (considered sleep of ≤6 h in duration [95]) and adverse cardiometabolic outcomes including obesity, hypertension, poor glucose regulation, and insulin resistance [19,95,96,97,98,99,100]. Importantly, extended sleep (i.e., sleep >9 h) is also associated with adverse cardiometabolic health effects [101,102], such as increased blood pressure [103,104], and this could be a bi-directional relationship, with long sleep a symptom of CVD [105]. This suggests a U-shape relationship between sleep and cardiometabolic health, with 7–9 h considered an optimum amount for favourable cardiometabolic outcomes [102]. Ensuring adequate sleep duration (i.e., 7–9 h per 24 h) is therefore a key strategy to reduce the risk of CVD.

Inadequate sleep is a common outcome of shift-work schedules [37]. Many shift workers, particularly those engaged in night work, experience some degree of circadian disruption [9,106]. This is thought to be a major contributor to the prevalence of cardiometabolic issues in shift workers, such as elevated post-prandial glucose levels, obesity, and CVD in the long-term [9,19]. Several systematic reviews have highlighted the increased risk of adverse cardiovascular outcomes and CVD in shift workers, particularly individuals undergoing night-shift work [107,108,109], further demonstrating the link between circadian disruption and CVD.

## 4. Inadequate Sleep Mediates the Relationship between Time-Restricted Eating and Cardiovascular Outcomes

Since risk factors for CVD include multiple lifestyle factors, it is paramount that interventions adopt a multi-faceted approach to combat the disease [110]. The relationship between sleep and eating patterns is well-established, with recent reviews demonstrating a link between nutrition and both sleep quality and quantity in adults [61,62]. Research has also identified a relationship between food intake and inadequate sleep, with shorter sleep duration leading to increased energy intake [56,60,111,112]. Furthermore, there is a relationship between food intake and some sleep-related disorders. For example, higher intake of red/processed meat and lower diet quality was reported in a sample of females diagnosed with obstructive sleep apnoea [113]; increased intake of fatty acids was found in a group of overweight and obese men with obstructive sleep apnoea compared to obese and overweight men without obstructive sleep apnoea [114]; and higher consumption of trans fats and sodium, in addition to a lower intake of vegetables, was found in a sample of men with probable insomnia compared with men without probable insomnia [115]. Additionally, the timing of sleep can also influence eating patterns. For example, individuals who sleep at times other than at night (as a result of shift work) report having to rearrange their eating patterns to accommodate sleep [55]. This may require eating before and/or after a day time sleep (i.e., late at night or early in the morning [55]) when the body is not in an optimal state to support digestion [14]. The timing of eating and sleeping can also affect the diversity and functioning of the gut microbiota, which is the population of microbes residing in the intestinal tract [116]. This is an additional link between sleep, eating, and cardiometabolic disorders [117], as changes to meal and sleep timing can disrupt the diurnal variations of the gut microbiome, which is associated with disruptions to processes such as energy absorption, lipid metabolism, and the production of short-chain fatty acids [118]. Several reviews have highlighted the link between the disrupted gut microbiome and cardiometabolic disorders [116,117,118].

**Figure 3:**
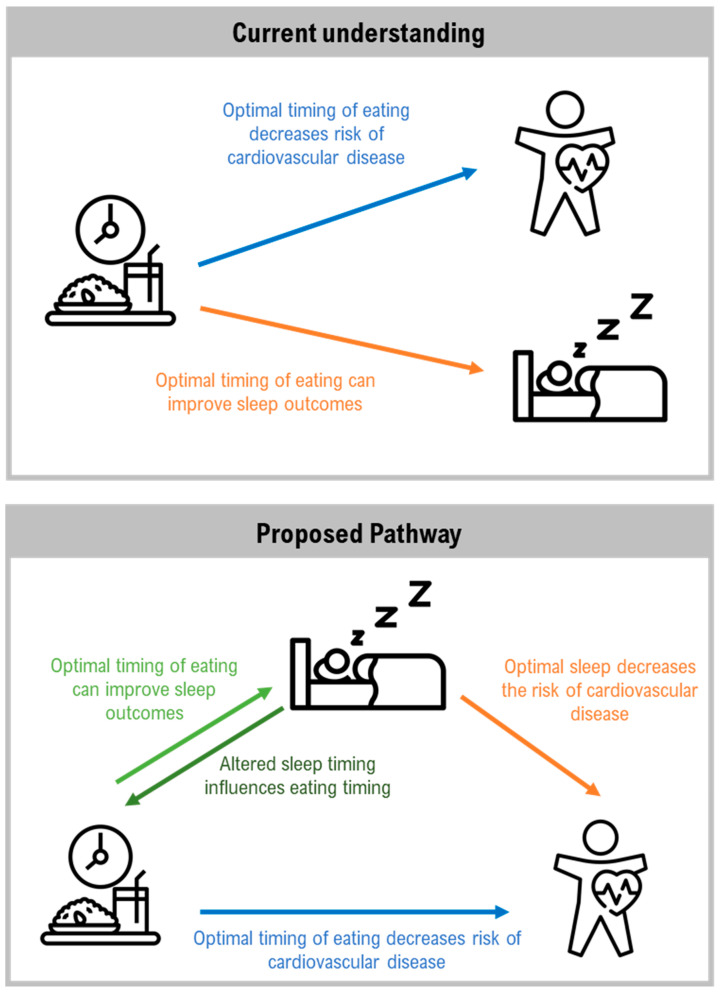
Current understanding of the relationship between timing of eating, sleep, and cardiovascular disease (top), and the proposed model linking timing of eating, sleep, and cardiovascular disease. Individual icons sourced from The Noun Project: Sleep by Sumit Saengthong from NounProject.com; Rice by ic2icon from NounProject.com; and Heart Disease by Lars Meiertoberens from NounProject.com.

As previously discussed, both eating and sleep are key cues for circadian alignment and are independently associated with an elevated risk of CVD [63,76,80,104,119]. Thus, exploring the interaction between sleep and eating behaviours is critical. Chrononutrition is a multi-disciplinary field aimed at understanding how the timing of food intake may impact health [44]. The two main functions of chrononutrition, as argued by Oda [120], are understanding the impact of meal timing on health and how meal timing entrains our body clock, including the sleep–wake system; thus, TRE as a strategy to improve health is an important part of the chrononutrition field. To date, limited research has been conducted on TRE and sleep, with studies largely focused on the effect of TRE on sleep outcomes. Indeed, a recent review by McStay et al. [53] identified eight studies that reported sleep-related outcomes after a period of TRE or intermittent fasting. Many of these studies had CVD risk factors as primary outcomes, such as glucose metabolism [121], body weight [47], and blood pressure [51], with sleep-related factors as a secondary outcome. Furthermore, by including sleep as an outcome variable (rather than a covariate), the influence of inadequate sleep on cardiometabolic outcomes is not considered. This is of concern given that inadequate sleep negatively affects the same cardiometabolic outcomes that TRE has been shown to improve [8,35]. We propose that sleep is considered a mediator, whereby inadequate sleep mediates the relationship between TRE and CVD (Figure 3). For example, if eating patterns are optimised with TRE but individuals experience inadequate sleep, then some level of circadian disruption is still likely. The benefits to health may therefore not be as robust. This has important implications for health interventions, as currently TRE is marketed as a relatively approachable eating strategy for improved health [45,77,78,84]. However, given the prevalence of inadequate sleep experienced by the general public, without providing concurrent advice on improving sleep, limited benefits to cardiometabolic health may be achieved from employing TRE in isolation.

## 5. Proposing a New Research Agenda

In this review, we have proposed a model suggesting poor sleep as a mediator in the relationship between TRE and cardiovascular health such that optimal timing of both sleep and eating are needed for the most favourable health outcomes. There are several key areas of investigation that are critical to testing this model. Broadly, this includes suggestions for study designs, outcomes measures, and populations investigated.

### 5.1. Study Design

To date, studies investigating TRE with sleep measures have been conducted in field settings [53] where there are clear limitations to internal validity related to extraneous variables (e.g., physical activity [110] and dietary behaviours [68]). We propose that future research should first employ rigorously controlled laboratory protocols [86,122,123] to investigate the effects of TRE and sleep duration on cardiovascular outcomes. In controlled laboratory settings, the window of eating can also be altered while keeping other variables constant. This will support an understanding of the relationship between TRE and cardiovascular outcomes, with and without sleep as a mediator. For example, comparing the impact of different eating windows (e.g., 14 h vs. 10 h eating window) and inadequate sleep (e.g., 5 h) vs. optimal sleep (e.g., 9 h).

TRE may be considered a straightforward approach as it does not require high levels of nutrition literacy to implement, nor changes to the types/amounts of food eaten [124]. However, behaviour change can be difficult [93] and both sleep and eating are influenced by a number of external factors (e.g., work, family, and stress) [110]. Therefore, the feasibility of adopting TRE while also targeting sleep timing needs to be determined with field studies. As a first step, investigating the impact of TRE on cardiovascular outcomes in individuals who experience inadequate compared to adequate sleep is required. The efficacy of TRE to influence cardiovascular outcomes, under intervention conditions aimed at improving sleep timing (e.g., facilitation of sleep education sessions), could then be investigated. It is important to note that a standardised methodology for implementing TRE and measuring both adherence and outcomes is needed as a matter of priority for future research [78]. Understanding the impact of TRE long-term is also important as the recent review by Gabel et al. [45] found that the longest study on TRE and cardiometabolic measures was conducted for 16 weeks. Longer studies will be crucial to understand the sustainability of TRE and whether the positive effects of TRE can be maintained long-term.

### 5.2. Outcome Measures

For consistency with the current literature, future research should continue to use several measures of cardiometabolic health including body weight, body composition, blood pressure, plasma lipids, inflammatory markers, glucose metabolism, and markers of oxidative stress. These are commonly used to investigate the impact of TRE on cardiometabolic health [12,42,45,65,121,125]. Conversely, a high degree of heterogeneity exists for the measures employed in previous studies investigating TRE and sleep outcomes [53], with many of the measures not validated. Changes in sleep as a result of TRE have been measured using a mixture of subjective (e.g., sleep diary [46] and Pittsburgh Sleep Quality Index [47,84,126]) and objective (e.g., Accelerometer [47,121]) assessment techniques. Future research utilising laboratory protocols should include polysomnography (PSG), the gold-standard in the objective measurement of sleep [127]. PSG assesses sleep via electrodes placed at several sites on the scalp (typically the frontal, central, and occipital brain regions) to record brain activity [127]. This allows sleep architecture to be assessed, which can be influenced by aspects of cardiovascular health such as glucose metabolism [128]. Finally, given that there are many external factors influencing both sleep and eating, qualitative methodological approaches that permit the exploration of barriers and enablers to implementing TRE are also needed.

### 5.3. Populations

There are several suggestions for populations to target in future research. A key recommendation from the review on intermittent fasting, TRE, and sleep by McStay et al. [53] was that longer-term trials with larger sample sizes are required as sample sizes in the eight studies identified in the review ranged from *n* = 8 to *n* = 116. Sample size is a limiting factor to the statistical power of studies and the ability to detect subtle differences in outcomes based on sleep or TRE. Future investigations must be appropriately powered a priori to ensure that adequate statistical analyses can be conducted.

Specific populations such as shift workers should be targeted in future research. As discussed throughout this review, shift workers experience chronic circadian disruption and have both sleep and eating patterns that vary across a 24 h period [55,73]. As such, they are a population at high risk of developing CVD [54] and understanding the effects of TRE combined with interventions aimed at optimising sleep timing is crucial for this group. While the impact of improved sleep timing and interventions for optimising sleep timing (e.g., strategic light exposure and napping [54]) have been investigated in shift workers, the feasibility and efficacy of TRE for shift workers is unknown. This area of inquiry is currently being investigated by Manoogian et al. [48]; however, it is important to note that sleep parameters are only being measured as a secondary outcome and analysed based on the influence of TRE.

Investigating the impact of TRE and sleep timing in individuals of varying chronotypes is also an important consideration for future research [53]. An individual’s chronotype reflects their personal circadian preference, with people typically categorised as either morning or evening types based on the timing of their sleep–wake cycle [129]. Thus far, chronotypes in relation to the timing of eating and cardiometabolic outcomes have only been assessed in young adults [130]. Given that a mismatch between chronotype and sleep timing can lead to circadian disruption and is a risk factor for CVD [6,131,132], this is worthy of attention. Furthermore, given that chronotypes can be an indicator of biological timing [129], an effort to use TRE to minimise circadian misalignment would need to do so relative to a person’s body clock rather than to the absolute clock time. There is also evidence that chronotype can influence the timing of eating, with evening chronotypes associated with consuming larger meals later in the day [133]. Therefore, it may be that the feasibility and effectiveness of TRE differs based on chronotype.

Lastly, given the association between diet quality, timing, and sleep disorders [113,114,134,135], understanding the impact of TRE for those diagnosed with sleep disorders is important. Furthermore, sleep disorders are associated with an increased risk of CVD [136,137], therefore, understanding whether TRE reduces the risk of CVD in this population could be a key strategy for reducing the burden of CVD.

## 6. Conclusions

To reduce the burden of cardiovascular disease, there is a need to optimise the timing of both eating and sleep. Given that the timing of eating and sleep are both key aspects of our circadian system and both target the same cardiometabolic parameters, this review has argued that they must be considered together to achieve optimal cardiometabolic outcomes. In this review, we proposed a novel approach to future research, with suggestions for study designs, outcome measures, and populations, to investigate the relationship between the timing of eating and sleep on cardiometabolic health. The goal of this research agenda is to inform interventions aimed at reducing the current burden of cardiovascular disease.

## Data Availability

Not applicable.
